# Development and Characterization of Sustainable Biocomposites from Wood Fibers, Spent Coffee Grounds, and Ammonium Lignosulfonate

**DOI:** 10.3390/polym17192589

**Published:** 2025-09-24

**Authors:** Viktor Savov, Petar Antov, Alexsandrina Kostadinova-Slaveva, Jansu Yusein, Viktoria Dudeva, Ekaterina Todorova, Stoyko Petrin

**Affiliations:** 1 Faculty of Forest Industry, University of Forestry, 1797 Sofia, Bulgaria; yusein@ltu.bg (J.Y.); v.dudeva@ltu.bg (V.D.); 2 Faculty of Ecology and Landscape Architecture, University of Forestry, 1797 Sofia, Bulgaria; aslaveva@ltu.bg (A.K.-S.); etodorova@ltu.bg (E.T.); 3 Faculty of Chemical Technologies, University of Chemical Technology and Metallurgy, 1757 Sofia, Bulgaria; stpetrin@uctm.edu

**Keywords:** biocomposite, spent coffee grounds, lignosulfonate, wood fibers, physical and mechanical properties, thermogravimetric analysis

## Abstract

Coffee processing generates large volumes of spent coffee grounds (SCGs), which contain 30–40% hemicellulose, 8.6–13.3% cellulose, and 25–33% lignin, making them a promising lignin-rich filler for biocomposites. Conventional wood composites rely on urea-formaldehyde (UF), melamine–urea–formaldehyde (MUF), and phenol–formaldehyde resins (PF), which dominate 95% of the market. Although formaldehyde emissions from these resins can be mitigated through strict hygiene standards and technological measures, concerns remain due to their classification as category 1B carcinogens under EU regulations. In this study, fiber-based biocomposites were fabricated from thermomechanical wood fibers, SCGs, and ammonium lignosulfonate (ALS). SCGs and ALS were mixed in a 1:1 ratio and incorporated at 40–75% of the oven-dry fiber mass. Hot pressing was performed at 150 °C under 1.1–1.8 MPa to produce panels with a nominal density of 750 kg m^−3^, and we subsequently tested them for their physical properties (density, water absorption (WA), and thickness swelling (TS)), mechanical properties (modulus of elasticity (MOE), modulus of rupture (MOR), and internal bond (IB) strength), and thermal behavior and biodegradation performance. A binder content of 50% yielded MOE ≈ 2707 N mm^−2^ and MOR ≈ 22.6 N mm^−2^, comparable to UF-bonded medium-density fiberboards (MDFs) for dry-use applications. Higher binder contents resulted in reduced strength and increased WA values. Thermogravimetric analysis (TGA/DTG) revealed an inorganic residue of 2.9–8.5% and slower burning compared to the UF-bonded panels. These results demonstrate that SCGs and ALS can be co-utilized as a renewable, formaldehyde-free adhesive system for manufacturing wood fiber composites, achieving adequate performance for value-added practical applications while advancing sustainable material development.

## 1. Introduction

Concurrently, agricultural residues such as spent coffee grounds (SCGs) are receiving increased attention due to their high availability and rich organic composition, including lignin, cellulose, polyphenols, and sugars [1,2,3,4,5]. Several studies [6,7,8] have highlighted the potential of SCG valorization in material production, particularly for polymer composites, biosorbents, and biofuels. Their binding behavior is primarily attributed to the presence of natural sugars and hydroxyl groups, which may contribute to cross-linking and water resistance. Coffee is among the most widely traded agricultural products, with global consumption continuing to increase and projected to grow by approximately 4% annually between 2024 and 2028 [9]. Brewing extracts only ~0.2 wt% of the bean; the remaining biomass, including SCGs, together with husks, pulp, and silverskin, constitutes the bulk of the coffee waste stream. In 2023/2024, approximately 11 million tonnes of SCGs were generated worldwide. On average, each cup of coffee produces ~10 g of SCG; a single coffee chain in Poland (100 cafés) was reported to generate ~180 t yr^−1^, with national production reaching ~120,000 t yr^−1^ [9,10,11]. SCGs are composed of 30–40% hemicellulose, 8.6–13.3% cellulose, and 25–33% lignin, along with polyphenols, lipids, proteins, and minerals [12,13,14,15]. Their lignin content contributes hydrophobic fatty groups that modify polymer processing behavior [11]. These chemical constituents have encouraged the use of SCGs as fillers in various polymers, e.g., polylactide (PLA), bio-based polyethylene (Bio-PE), polyhydroxyalkanoates (PHAs), and bio-polyamides, where they enhance stiffness and thermal stability but may reduce tensile strength at high loading [11,16,17,18,19,20].

Commercial wood-based composites are typically bonded with UF, melamine-UF, or phenol-formaldehyde (PF) resins; these petroleum-derived adhesives account for >95% of the adhesive market in engineered wood products [21,22,23,24]. However, these formaldehyde resins emit free formaldehyde, classified in 2004 by the International Agency for Research on Cancer (IARC) as a Group 1 human carcinogen, and were reclassified in 2015 under EU legislation as category 1B carcinogens [25]. In response, emissions regulations have become increasingly stringent (e.g., Germany’s E0.5 class limits free formaldehyde to ≤0.05 ppm) [26]. Consequently, the development of formaldehyde-free adhesive systems has become a major research and industrial priority.

Lignin is the second most abundant natural polymer after cellulose, but only a small fraction is valorized into value-added products [27,28,29,30,31,32,33]. Among its derivatives, lignosulfonates (LS), by-products of the sulfite pulping process, are water-soluble and reactive due to sulfonic groups, making them attractive as renewable binder systems for wood composites [23,34,35,36,37,38,39,40,41,42,43,44,45]. Nevertheless, unmodified LS generally display limited reactivity and low bonding efficiency, which restricts their independent use and motivates research on combined or modified adhesive systems [46,47,48,49].

SCG has been explored as a filler in thermoplastics, where it enhances stiffness and UV resistance but often decreases tensile strength due to poor fiber–matrix adhesion [11]. Chemical modifications, such as alkali and silane treatments, have improved compatibility with polyhydroxyalkanoates [11]. In contrast, oil-extracted SCG has been reported to increase the flexibility and biodegradability of PLA films [9]. However, the combined use of SCG and LS as a binder matrix for wood fibers has not been systematically investigated. We hypothesize that the sugars and hydroxyl functionalities in SCGs, combined with the sulfonic acid groups in LS, may interact with cellulose fibers through hydrogen bonding and limited cross-linking, providing the basis for formaldehyde-free fiber-based panels. To the best of our knowledge, this synergistic use of SCG and LS has not been previously reported. At the same time, potential challenges, such as the hygroscopic character of LS and the relatively low intrinsic strength of SCGs, must be considered when evaluating the feasibility of this approach. The present study, therefore, investigated the influence of SCG and LS content on the physical, mechanical, and thermal properties of wood fiber biocomposites and evaluated their performance against conventional UF-bonded panels.

## 2. Materials and Methods

The primary raw materials used for biocomposite fabrication were spent coffee grounds (SCGs), ammonium lignosulfonate (ALS), and thermomechanical fibers. SCG was collected from local coffee shops after different brewing methods to reflect the heterogeneity of real waste streams. While this approach ensured representative heterogeneity, it also introduced variability in chemical composition, which may limit the strict repeatability of the experiments. This limitation is acknowledged as inherent to valorization studies relying on real industrial by-products. Upon collection, the SCG had a moisture content of ~61% and was prone to microbial contamination. Drying was therefore carried out under controlled indoor conditions (≈20 °C) in the Laboratory of Biocomposite Materials, University of Forestry (Sofia, Bulgaria). The material was spread in thin layers and stirred periodically for 2–3 weeks until the moisture content decreased below 10%. After drying, the SCG was stored in sealed, oxygen-free containers to prevent rehydration and microbial growth.

Ammonium lignosulfonate (ALS) was provided in the form of a concentrated aqueous solution of about 50% solids and was employed as the bio-based binder. The main characteristics of ALS were as follows: 48.6% dry matter, 4.1% ammonium, 0.1% sodium, 6.8% sulfur, approximately 20% sugars, a viscosity of 400 cps at 25 °C, pH 4.5, specific gravity of 1.220 g cm^−3^, and a boiling point near 104 °C.

Thermomechanical fibers were obtained on an industrial scale by the Asplund process using a Defibrator L56 unit (Valmet, Stockholm, Sweden) at the Kronospan Bulgaria PLC plant (Veliko Tarnovo, Bulgaria). The fiber furnish was composed of about 40% hardwoods—European beech (*Fagus sylvatica*) and Turkish oak (*Quercus cerris*)—and 60% softwoods, primarily Norway spruce (*Picea abies*) and Scots pine (*Pinus sylvestris*). The fibers, with an initial moisture content of around 10%, were subsequently applied as the reinforcing component in the composite boards.

The preliminary development of the biocomposite was focused on evaluating the inherent adhesive capacity of SCGs. In the first trial, moistened SCG was hot-pressed at 160 °C for 2 min without the addition of any binder. Partial adhesion was achieved, likely due to the caramelization of residual sugars; however, the product was brittle and deteriorated over time as hydroxyl bonds hydrolyzed. In a second trial, SCG was combined with thermomechanical fibers before pressing under the same conditions. This increased bonding and slightly reduced brittleness; however, the material still lacked sufficient rigidity for structural applications. In the third formulation, ALS was incorporated together with SCGs and fibers. The resulting composite exhibited significantly improved stiffness, stronger chemical bonding, and greater moisture stability. Additionally, the presence of lignosulfonate imparted a mild surface gloss, attributed to its sugar content and thermoplastic behavior during hot pressing.

**Figure 1:**
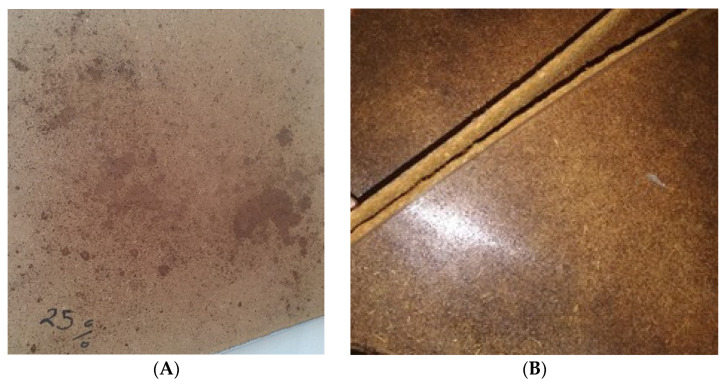
Non-uniform distribution of SCG–ALS bio-adhesives at total concentrations below 40% (**A**), and delamination occurring at a concentration of 80% (**B**).

Based on the preliminary results, the ternary system of SCGs, ALS, and thermomechanical fibers was selected for optimization. The binder content, expressed as the combined mass of sum of SCG and ALS relative to oven-dry fibers, was varied from 20% to 80%. At 20% binder content (10% SCG and 10% ALS), non-uniform dispersion was observed, with localized binder accumulation and variable surface gloss. Increasing the binder content to 25% improved uniformity when mixed for three minutes in a blender and pressed at 1.1 MPa and 150 °C for 15 min, although small localized spots persisted. At 30% binder content, a split lignosulfonate approach was applied: half of the ALS was sprayed onto the fibers, while the other half was mixed directly with SCG prior to blending. The panels pressed under these conditions (1.8 MPa, 150 °C, 16 min) showed improved gloss but still exhibited areas with excess binder. Binder levels between 40% and 75% progressively enhanced surface finish and cohesion, and pre-drying fibers at 40% binder content further increased uniformity. However, at 80% binder content, the panels exhibited poor internal cohesion and delaminated upon removal from the press (Figure 1), indicating that excessive binder hindered fiber interlocking. From these trials, 75% binder was established as the upper practical limit for stable composite formation. Accordingly, five final formulations, i.e., 40%, 50%, 60%, 70%, and 75%, were selected for detailed evaluation.

The experimental plan is summarized in Table 1. All panels were manufactured at a target density of 750 kg·m^−3^, with dimensions of 400 mm × 400 mm × 6 mm, and were hot-pressed under controlled temperature and pressure regimes depending on binder content. This target value corresponds to the typical density range of standard MDF panels (720–780 kg·m^−3^) used for interior applications and was selected to ensure comparability with industrial products.

For comparison, reference panels bonded with 10% urea-formaldehyde (UF) resin (based on oven-dry fiber mass) were produced under identical pressing conditions and used as controls. These panels represent the conventional bonding system in fiberboard production and served as benchmarks for evaluating the performance of the laboratory-fabricated SCG–ALS composites.

The determination of physical and mechanical properties of the manufactured panels followed the respective European standards. Density was measured in line with EN 323 [50]. Water absorption (WA) and thickness swelling (TS) after 24 h submersion in water were assessed according to EN 317 [51]. Bending strength parameters, i.e., modulus of rupture (MOR) and modulus of elasticity (MOE), were determined following EN 310 [52], and the internal bond (IB) strength was evaluated in accordance with EN 319 [53]. Before testing, all specimens were conditioned in a climate of 20 ± 2 °C and 65 ± 5% relative humidity until they reached equilibrium moisture content. For each formulation and property, eight replicate specimens were tested in accordance with EN standards to ensure statistical reliability.

Mechanical properties were determined using a universal testing machine (UTM, model WDW-50E, HST, Jinan, China). The density profile of the panels was measured with a GreCon DENSITYPROFILER (Fagus-GreCon Greten GmbH & Co. KG, Alfeld, Germany). Thermogravimetric (TGA/DTG) analysis was performed to evaluate the thermal stability and combustion behavior of the fabricated composites. The objective was to assess their potential fire resistance and durability, which are critical factors for practical applications of wood-based panels. TGA/DTG were carried out on a STA PT1600 TG-DTA/DSC simultaneous thermal analyzer (LINSEIS Messgeräte GmbH, Selb, Germany).

The experimental data were subsequently processed and statistically evaluated using Microsoft Excel (Microsoft Office 365), which was employed to calculate average values and standard deviations and to generate the corresponding graphs.

## 3. Results and Discussion

**Figure 2:**
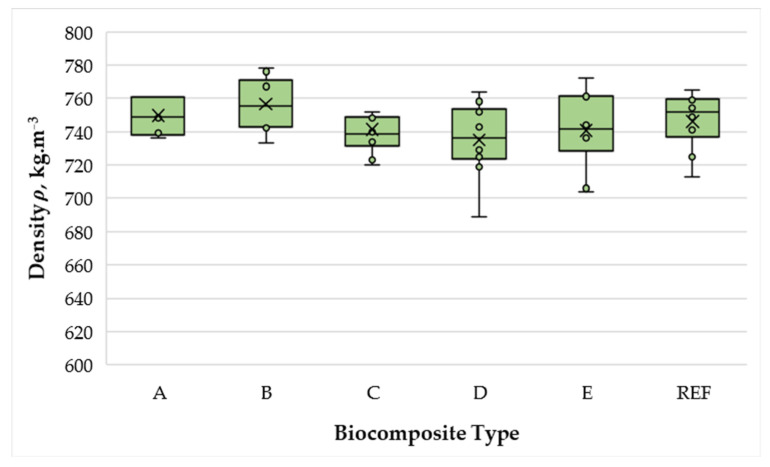
Density of biocomposite panels with varying proportions of wood fibers, spent coffee grounds, and ammonium lignosulfonate. A–E correspond to panels bonded with increasing binder content (40, 50, 60, 67, and 75 wt% SCG + ALS relative to oven-dry fibers, respectively). REF denotes the reference panel bonded with 10 wt% urea–formaldehyde (UF) resin.

All biocomposites exhibited densities of 735–756 kg·m^−3^ (variation ±2.8%), indicating that the applied press regime was effective in achieving the target density. The narrow variation indicates that differences in physical and mechanical performance can be attributed primarily to formulation and processing parameters rather than density deviations (Figure 2).

**Figure 3:**
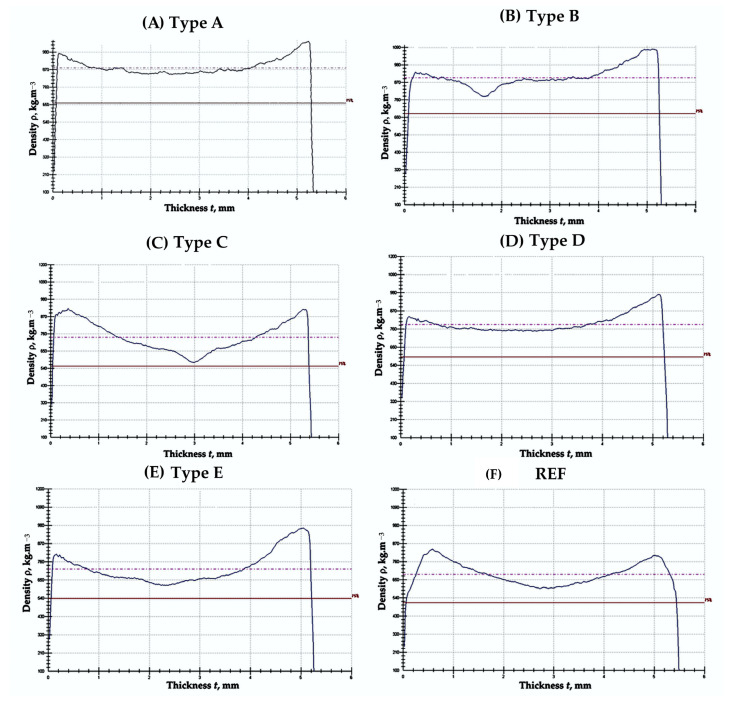
Density profile of biocomposite panels with varying proportions of wood fibers, coffee grounds, and ammonium lignosulfonate: (**A**) 40%—total content of SCG and ALS at 40% relative to the absolutely dry fibers; (**B**) 50%—total content of SCG and ALS at 50% relative to the absolutely dry fibers; (**C**) 60%—total content of SCG and ALS at 60% relative to the absolutely dry fibers; (**D**) 70%—total content of SCG and ALS at 70% relative to the absolutely dry fibers; (**E**) 75%—total content of SCG and ALS at 75% relative to the absolutely dry fibers; (**F**)—reference panel with a 10% content of UF resin relative to the absolutely dry fibers.

Density profiles revealed higher density near the bottom surface of all panels, attributed to partial sedimentation of SCG during pressing. The top–bottom surface density difference increased from ~7% (40% binder) to ~20% (75% binder). Average density variation across the thickness remained below 15%, comparable to or lower than that of the UF-bonded control. SCG sedimentation could be mitigated by increasing binder viscosity or applying pre-compression (Figure 3).

Similar asymmetric density profiles have been reported for fiberboards with high lignin-based binder contents, especially when binder viscosity is low [21]. Panels bonded with LS–UF adhesive systems have shown a difference of up to 25% between face and core densities under comparable hot-pressing conditions [27]. The present results show better uniformity than some earlier reports, likely due to the finer SCG particle size and the split addition of lignosulfonate during mixing.

**Figure 4:**
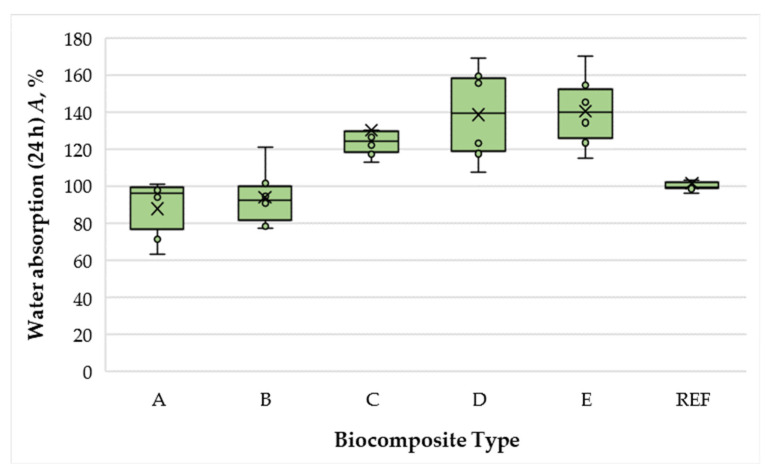
Water absorption of biocomposite panels with varying proportions of wood fibers, spent coffee grounds, and ammonium lignosulfonate. A–E correspond to panels bonded with increasing binder content (40, 50, 60, 67, and 75 wt% SCG + ALS relative to oven-dry fibers, respectively). REF denotes the reference panel bonded with 10 wt% urea–formaldehyde (UF) resin.

Water absorption (WA) results of the laboratory-fabricated biocomposite panels are presented in Figure 4.

Under the conditions of this study, WA values ranged from 97.91% to 138.56%. Increasing the total SCG–ALS content from 40% to 50% reduced WA by a factor of 1.07, which can be attributed to the higher binder content in the biocomposites. Notably, panels containing 40% and 50% SCG–ALS exhibited lower WA than the UF-bonded control (10% UF resin), indicating satisfactory water resistance at these levels. However, increasing the binder content beyond 50% led to a pronounced deterioration in water resistance. At 60% SCG–ALS, WA increased by a factor of 1.41 relative to the 50% formulation, while the 75% formulation exhibited 1.53 times higher WA. This effect can be attributed to the excess moisture introduced at high SCG–ALS contents, which is difficult to remove during hot pressing and may lead to slight thermal degradation of the biocomposite constituents. Furthermore, increasing the matrix phase (SCG and ALS) above 50% reduces the proportion of reinforcing wood fibers, thereby compromising dimensional stability.

**Figure 5:**
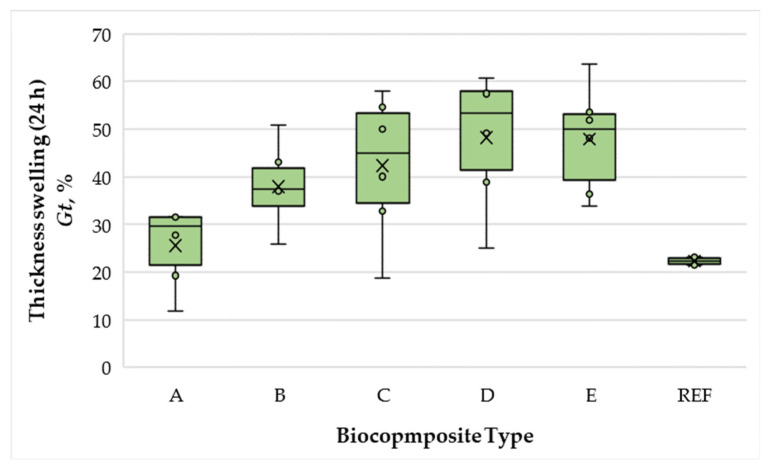
Thickness swelling of biocomposite panels with varying proportions of wood fibers, spent coffee grounds, and ammonium lignosulfonate. A–E correspond to panels bonded with increasing binder content (40, 50, 60, 67, and 75 wt% SCG + ALS relative to oven-dry fibers, respectively). REF denotes the reference panel bonded with 10 wt% urea–formaldehyde (UF) resin.

The variation in thickness swelling (TS) of the bicomposite panels is presented in Figure 5.

Under the conditions of this study, TS values of the biocomposite panels ranged from 22.29% to 48.22%. Increasing the total SCG–ALS content from 40% to 50% produced no significant change in TS (difference of 4.6%, within statistical error). However, further increases in binder content led to progressively higher swelling, with the 75% formulation exhibiting 1.56 times greater TS compared with the 40% formulation. None of the biocomposites met the EN 622-5 [54] requirement for MDFs (TS values ≤ 30%), although both the 40% and 50% SCG–ALS panels showed lower TS and WA values than the UF-bonded control, indicating that moderate binder levels enhance water resistance. Nevertheless, the control panel bonded with 10% UF resin still outperformed all SCG–ALS formulations, showing 1.38 times lower TS than the best-performing biocomposite. It should be noted, however, that the WA values of the best-performing formulations (40–50% SCG–ALS) still approached 100%, which is far above the EN 622-5 [54] requirement for MDFs. This limited water resistance can be attributed in part to the absence of paraffin emulsion, commonly used as a hydrophobic additive in industrial MDF production, as well as to the fact that commercial panels are typically bonded with MUF resins or UF resins modified with up to 10% melamine. Consequently, while moderate SCG–ALS contents improved dimensional stability compared with the UF control, the water resistance remains insufficient for load-bearing applications. Further improvements, such as chemical modification of ALS or the use of suitable crosslinkers, are necessary to meet practical requirements.

Similar trends have been observed in prior research, where LS-based adhesives require moisture to activate hydrogen bonding. When LS content exceeds ~30% of UF resin, the internal bond strength of boards typically decreases due to the low reactivity of unmodified LS toward formaldehyde and the introduction of excess moisture [21]. To improve water resistance, chemical modifications such as methylolation or phenolation of LS and the use of crosslinkers (e.g., pMDI or furfuryl alcohol) have been proposed [27].

**Figure 6:**
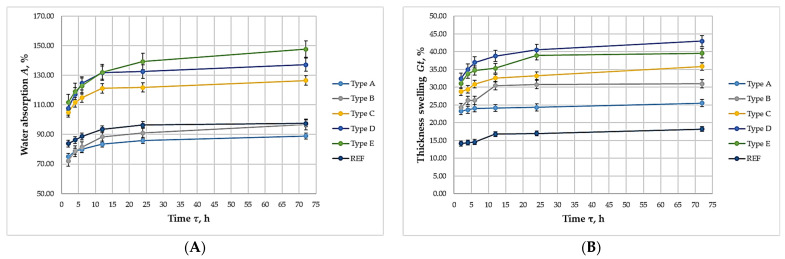
Kinetics of water absorption and thickness swelling of biocomposite panels with varying proportions of wood fibers, spent coffee grounds, and ammonium lignosulfonate: (**А**) Water absorption; (**B**) Thickness swelling.

Figure 6 presents the kinetics of WA and TS of the laboratory-fabricated biocomposites.

The data indicate that all biocomposites exhibited WA and TS kinetics comparable to those of the UF-bonded control panel. This suggests that the biocomposites developed relatively water-resistant bonds and may be suitable for applications involving exposure to moisture. In practice, most water uptake and swelling occurred within the first 12 h of immersion, after which no significant changes were observed up to 72 h, indicating stabilization of the material properties.

**Figure 7:**
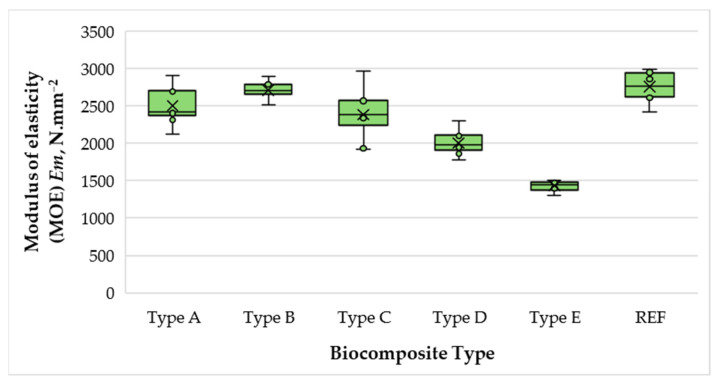
Modulus of elasticity (MOE) of biocomposite panels with varying proportions of wood fibers, spent coffee grounds, and ammonium lignosulfonate.

Figure 7 presents the variation in the modulus of elasticity (MOE) of the biocomposite materials depending on wood fibers, SCGs, and ALS content.

The MOE values of the biocomposites ranged from 1431 to 2707 N·mm^−2^. A notable trend was observed up to 50% SCG–ALS content (relative to oven-dry fibers), where MOE increased by 1.09 times compared to the 40% formulation. Beyond this level, MOE progressively declined by 13% at 60%, by 19% at 70%, and most drastically at 75%, where the bending stiffness dropped by 40% compared with the 50% formulation. This behavior can be attributed to the more uniform distribution of the matrix phase at 50%, which promotes stronger adhesion between binder and fibers, as well as the formation of additional hydrogen bonds facilitated by the higher sugar and hydroxyl content in SCGs. The improved stress transfer at this ratio likely reflects an optimal balance between the reinforcing fiber fraction and the matrix phase.

At binder levels above 50%, however, several detrimental effects become evident. Excessive binder leads to difficulty in removing steam–gas mixtures generated during pressing, which elevates local humidity and thermal conductivity. These conditions can induce partial thermal degradation of the lignocellulosic components, weakening the fiber network and reducing stiffness. Moreover, increasing the binder fraction simultaneously decreases the relative amount of reinforcing fibers, limiting the structural integrity of the composite. Consequently, while moderate SCG–ALS content (approximately 50%) enhances stiffness to levels comparable to those of UF-bonded panels for dry-use applications, excessive binder content (>50%) compromises panel rigidity and overall mechanical performance.

Markedly, at 50% SCG–ALS content, the biocomposite exhibited MOE values nearly identical to those of the UF-bonded control panel (difference of only 1.7%). Moreover, this formulation satisfied the EN 622-5 [54] requirement for general-purpose MDF panels, which specifies a minimum bending MOE of 2700 N·mm^−2^. Equally encouraging is that all biocomposites, fabricated without preliminary fiber drying, fulfilled or exceeded the EN 312 [55] requirements for type P2 particleboard (intended for interior use, including furniture in dry conditions), which require a minimum bending MOE of 1950 N·mm^−2^. In several cases, the measured values surpassed this threshold by a substantial margin, highlighting the potential of SCG–ALS binders to serve as viable, formaldehyde-free alternatives in value-added panel applications.

The observed optimum at 50% binder content also aligns with reports that moderate incorporation of lignin derivatives can improve bonding through enhanced hydrogen bonding and matrix uniformity, whereas excessive lignin may introduce excess moisture and promote thermal degradation during pressing, thereby reducing strength [26,56,57].

**Figure 8:**
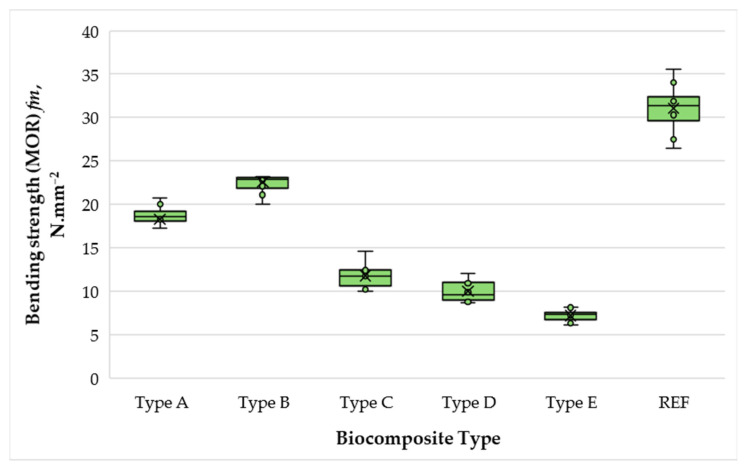
Bending strength (MOR) of biocomposite panels with varying proportions of wood fibers, spent coffee grounds, and ammonium lignosulfonate. A–E correspond to panels bonded with increasing binder content (40, 50, 60, 67, and 75 wt% SCG + ALS relative to oven-dry fibers, respectively). REF denotes the reference panel bonded with 10 wt% urea–formaldehyde (UF) resin.

The variation in the bending strength (MOR) of biocomposite materials depending on the content of wood fibers, SCGs, and ALS is presented in Figure 8.

With an increase in the matrix phase content from 40% to 50%, the MOR values improved from 18.33 to 22.55 N·mm^−2^, corresponding to a 1.23-fold increase. In contrast to the trend observed for MOE, however, MOR values decreased sharply when the matrix content was raised from 50% to 60%, with a 1.9-fold reduction. Further increases to 70% and 75% binder resulted in additional decreases of 1.18 and 1.39 times, respectively. This deterioration can be attributed to difficulties in evacuating steam–gas mixtures during pressing at high SCG–ALS contents, as well as to weaker bonding between SCG and ALS particles, which increasingly dominate the matrix when fiber reinforcement is reduced [58,59]. These findings are consistent with earlier reports, which show that partial substitution of UF with LS can increase MOR by 9–12% at 10–20% replacement; however, higher substitution levels generally reduce bending strength [21]. Similarly, boards produced with ALS–UF adhesives supplemented with small amounts of pMDI achieved MOR values comparable to UF-bonded panels [21]. In the present study, the composites with 50% binder content not only exceeded the EN 312 [55] requirement for type P2 particleboard (MOR ≥ 11 N·mm^−2^) but also met the EN 622-5 [54] requirement for MDFs in terms of MOE (≥2700 N·mm^−2^). Although the control UF panel exhibited higher MOR and MOE overall, the SCG–ALS biocomposites displayed markedly different failure behavior. Stress–strain curves revealed that the UF panel failed abruptly at <0.2% strain, typical of brittle fracture, whereas the biocomposites showed a more gradual stress decline with residual strength maintained up to ~2% strain. This ductile-like response reflects the cohesive character of lignin- and hydrogen-bonding networks within the SCG–ALS matrix, suggesting improved energy dissipation and toughness despite lower peak strength [60,61].

**Figure 9:**
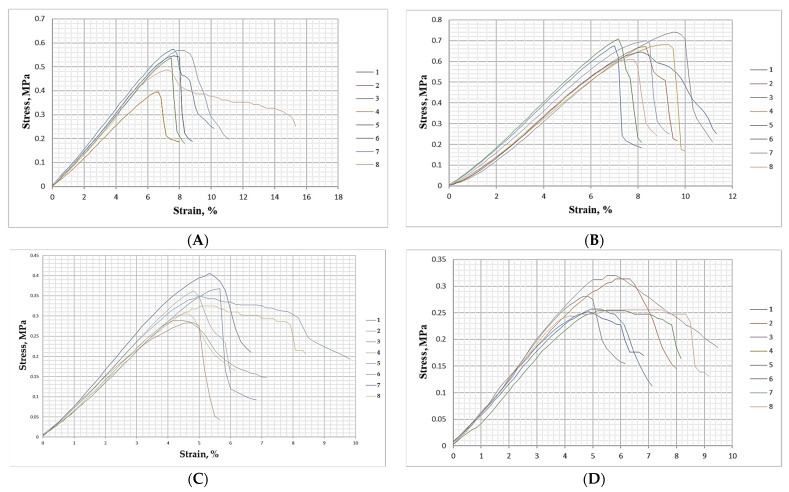

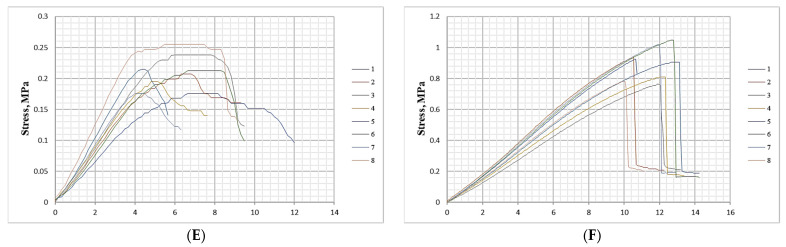
Deformation behavior at bending of biocomposite materials with different inclusions of wood fibers, spent coffee grounds and ammonium lignosulfonate: (**A**) 40%—total content of SCG and ALS at 40% relative to the absolutely dry fibers; (**B**) 50%—total content of SCG and ALS at 50% relative to the absolutely dry fibers; (**C**) 60%—total content of SCG and ALS at 60% relative to the absolutely dry fibers; (**D**) 70%—total content of SCG and ALS at 70% relative to the absolutely dry fibers; (**E**) 75%—total content of SCG and ALS at 75% relative to the absolutely dry fibers; (**F**)—reference panel with a 10% content of UF resin relative to the absolutely dry fibers.

The deformation behavior of the materials under bending is presented in Figure 9.

Despite the lower peak strength of the biocomposites composed of wood fibers, SCGs, and ALS compared with the control MDF panel manufactured with 10% urea–formaldehyde resin as binder, analysis of the stress–strain curves revealed a markedly higher residual strength for the biocomposites. In the UF-bonded MDF panels, deformation beyond 0.2% resulted in an ~80% drop in stress resistance, indicating almost instantaneous fracture typical of brittle failure and reflecting the limited elasticity of UF adhesive bonds. In contrast, the biocomposites exhibited a gradual decline in stress after the initiation of failure. On average, a 20% reduction in stress resistance corresponded to nearly 2% deformation, and in several cases, a plateau region was observed where the tensile strength did not decrease further immediately after fracture. This ductile-like response can be attributed to the cohesive bonding within the SCG–ALS matrix, which relies on both lignin–lignin and lignin–carbohydrate hydrogen bonds. Such interactions have been shown to contribute significantly to the toughness and cohesive strength of lignin-based systems [56,57,58,59,60]. The gradual stress decline observed here is also reminiscent of wet-processed hardboards, where lignin softening and hydrogen bonding dominate bonding mechanisms, leading to enhanced energy dissipation and toughness compared with synthetic UF systems [62,63]. These findings suggest that although SCG–ALS composites may not yet match UF-bonded panels in peak strength, their improved toughness and gradual failure mode provide important advantages in applications where resistance to critical fracture is desirable.

**Figure 10:**
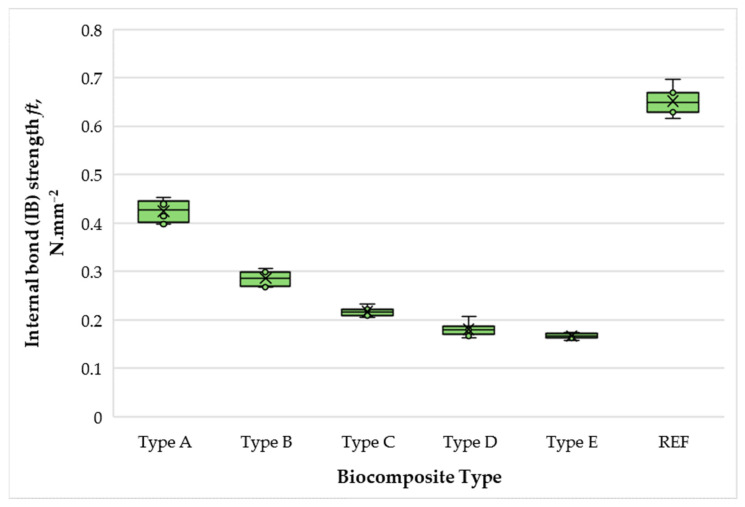
Internal bond (IB) strength of biocomposite panels with varying proportions of wood fibers, spent coffee grounds, and ammonium lignosulfonate. A–E correspond to panels bonded with increasing binder content (40, 50, 60, 67, and 75 wt% SCG + ALS relative to oven-dry fibers, respectively). REF denotes the reference panel bonded with 10 wt% urea–formaldehyde (UF) resin.

Figure 10 presents the variation in internal bond (IB) strength of biocomposite materials depending on the content of wood fibers, SCGs, and ALS.

The IB strength of the biocomposites varied from 0.29 to 0.65 N·mm^−2^ under the study conditions, representing a 2.24-fold difference across formulations. Increasing the total SCG–ALS content from 40% to 50% resulted in a 1.48-fold improvement in IB strength. Beyond this point, however, a sharp decline was observed, with IB decreasing by 1.97 times when binder content exceeded 50%. At 70% and 75% binder content, the differences in IB strength were relatively small (≤1.1-fold). This behavior can be explained by the homogenization of the matrix at a 50% binder content, where a higher concentration of SCG and ALS facilitates better adhesion and fiber encapsulation. However, the moisture content becomes excessive at higher binder levels, which cannot be effectively removed during hot pressing—conditions that have been shown to degrade board quality and internal strength in MDF systems [64]. Additionally, binder-rich compositions diminish the proportion of reinforcing wood fibers, disrupting the dense fiber network essential for cohesion [65].

As expected, none of the SCG–ALS biocomposites reached the IB strength of the UF-bonded control panel, nor did they meet the EN 622-5 [54] requirements for general-purpose MDFs. Nonetheless, the 50% SCG–ALS formulation exceeded the EN 312 [55] requirement for type P2 particleboard (intended for furniture production in dry conditions), indicating that such panels could be suitable for value-added, formaldehyde-free applications in non-structural interior uses.

These trends align with previous reports showing that LS adhesives generally yield lower IB strength than UF resins, mainly due to their limited reactivity. However, significant improvements in both IB strength and water resistance have been achieved when LS is chemically modified or supplemented with crosslinkers. For example, Hemmilä et al. fabricated particleboards with LS in the surface layers and pMDI in the core, achieving EN 312 type P1 performance at a low press factor of 7.5 s·mm^−1^ with E0.5-class emissions [27]. Similarly, Bekhta et al. reported that replacing 10–30% of UF resin with magnesium or sodium LS supplemented with pMDI produced panels with properties comparable to UF-bonded boards while markedly reducing formaldehyde emissions [49]. In contrast, LS–UF adhesives without crosslinkers typically show reduced MOR and IB, although formaldehyde emissions can decrease by up to 91% (MgLS) and 57% (NaLS) compared with conventional UF systems [21]. These findings highlight that while unmodified LS, such as ALS used in the present study, exhibit limitations in IB performance, targeted modifications or co-binders can substantially extend their application potential.

Our results confirm this trend: SCG–ALS panels demonstrated promising behavior, yet none of the formulations met the EN 622-5 [54] requirement for general-purpose MDFs in terms of IB strength. This limitation represents a major barrier for structural applications and underlines the need for further optimization. Potential strategies include chemical modification of ALS to increase reactivity or the incorporation of small amounts of crosslinkers such as pMDI or furfuryl alcohol. Therefore, while SCG–ALS adhesives show potential as formaldehyde-free binders, their use is currently restricted to non-structural interior products. Future developments must focus on improving bond performance to enable broader industrial applications.

**Figure 11:**
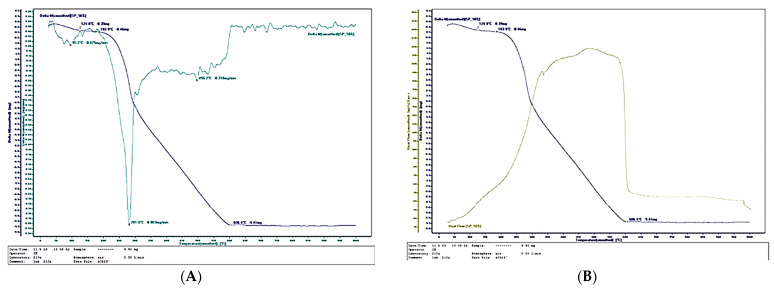
Thermal behavior of a bicomposite with 50% total content of SCG and ALS: (**A**) Thermogravimetry; (**B**) Differential scanning calorimetry.

**Figure 12:**
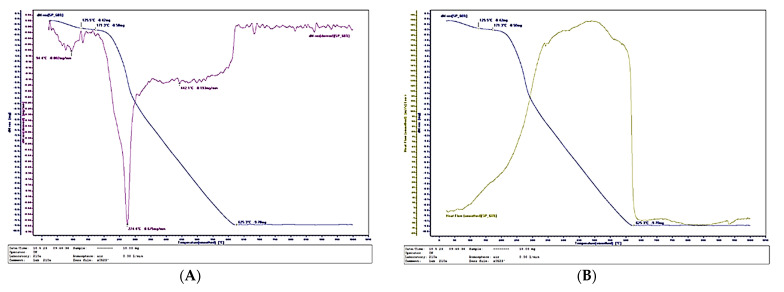
Thermal behavior of a biocomposite with 60% total content of SCG and ALS: (**А**) Thermogravimetry; (**B**) Differential scanning calorimetry.

**Figure 13:**
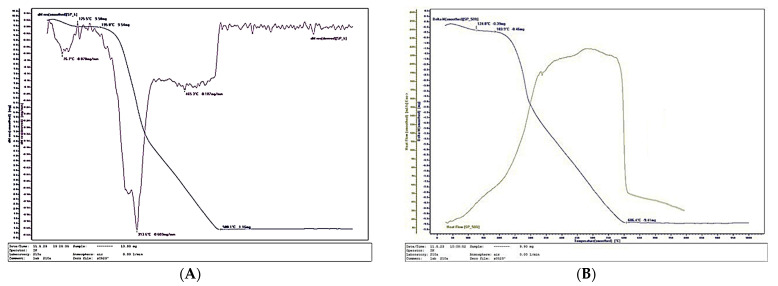
Thermal behavior of the control panel fabricated with 10% UF resin: (**А**) Thermogravimetry; (**B**) Differential scanning calorimetry.

Figure 11, Figure 12 and Figure 13 present the thermal analysis results of the SCG–ALS biocomposites compared with the UF-bonded control panel. The TGA/DTG data provide insight into thermal stability and combustion behavior, allowing evaluation of the composites’ potential in terms of fire resistance and long-term durability. This link to the research hypothesis is particularly relevant, since the feasibility of SCG–ALS as a bio-based adhesive system depends not only on mechanical performance but also on resistance to degradation under thermal stress.

The TGA/DTG curves clearly confirm the organic (bio-based) nature of the fabricated composites containing wood fibers, SCG and ALS. The amount of inorganic residue decreased with increasing binder content: 8.46% for the 40% SCG–ALS composite, 4.45% for the 50% composite, and only 2.88% for the 60% composite. In contrast, the UF-bonded control panels yielded a significantly higher inorganic residue of 11.57%. The inorganic inclusions in the SCG–ALS composites can be attributed primarily to the mineral constituents naturally present in the wood fibers [66,67].

Differential scanning calorimetry (DSC) further confirmed the influence of lignosulfonates as flame-retardant components. The phase transition (onset of volatilization into gaseous products) in the SCG–ALS biocomposites occurred in the range of 270–301 °C, depending on formulation, whereas the UF-bonded control panel exhibited a higher transition temperature of 313.6 °C. Although the biocomposites began to volatilize at slightly lower temperatures than the UF panel, their overall combustion behavior indicated improved fire resistance. Complete combustion of all organic constituents was observed at 595–625 °C for the biocomposites, compared with 580 °C for the UF control. The maximum rate of heat flow, an indicator of combustion rate, was significantly lower in the SCG–ALS composites: 192.356 mJ·s^−1^ (40% binder), 191.086 mJ·s^−1^ (50% binder), and 189.694 mJ·s^−1^ (60% binder), compared with 238.273 mJ·s^−1^ for the UF-bonded control panel. On average, the burning rate of the biocomposites was approximately 1.25 times lower than that of the UF control. Similarly, the total combustion time at a heating rate of 10 °C·min^−1^ was longer for the SCG–ALS composites (58 min at 40–50% binder, 62 min at 60% binder) compared with 54 min for the UF-bonded control, confirming slower thermal degradation.

These results align with the current literature, which indicates that lignosulfonates act as effective flame retardants by promoting char formation and improving thermal stability [21]. Recent studies have demonstrated that lignin-based additives can enhance fire resistance and reduce environmental impact in biocomposites [68,69,70]. Moreover, specific studies using LS with ammonium polyphosphate in IFR formulations report significant gains in char yield and thermal performance—such as lower heat release and delayed combustion—confirming the protective role of LS.

The results obtained demonstrate that combining SCGs and ALS provides a viable formaldehyde-free binder system for wood-based fiberboards. The optimum binder content (50%) yielded MOE and MOR values comparable to UF-bonded boards, while also exhibiting lower WA values and slower combustion. Increasing binder content beyond 50% proved unfavorable due to elevated moisture levels and reduced fiber fraction, highlighting the critical importance of optimizing the matrix–fiber ratio. These findings are consistent with industrial studies reporting that partial substitution of UF with 10–30% LS can maintain comparable mechanical properties, whereas pure LS adhesives generally fail to meet strength requirements [21]. The addition of crosslinkers such as pMDI has been shown to restore or even enhance mechanical performance while drastically reducing free formaldehyde emissions [21,27]. For instance, magnesium LS oxidized with hydrogen peroxide and crosslinked with 3% pMDI or with 2% pMDI plus 15% glucose achieved bending strengths of 0.38–0.41 N·mm^−2^ and formaldehyde emissions within E1/E0 limits [71]. Chemical modifications of LS, such as methylolation or phenolation, have also been reported to improve reactivity and water resistance [72]. In the present study, no synthetic crosslinkers were employed; however, performance could likely be further enhanced by introducing small amounts of pMDI or furfuryl alcohol.

From a circular economy standpoint, valorizing SCG as a lignin-rich binder closes the loop on coffee waste and reduces reliance on fossil-based resins. Building on prior uses of SCGs in biopolymers [9,11], this study demonstrates their feasibility in lignin-based MDFs for value-added wood composites.

## 4. Conclusions

This study demonstrated the feasibility of producing formaldehyde-free biocomposite panels from wood fibers, spent coffee grounds (SCGs), and ammonium lignosulfonate (ALS). The results confirmed that binder content is the key factor governing performance. A formulation with 50% SCG–ALS (relative to oven-dry fibers) provided an optimum balance, yielding MOE and MOR values comparable to UF-bonded MDF panels, while also offering improved water resistance and enhanced thermal stability. Below 40%, insufficient binder led to poor cohesion, whereas contents above 50% reduced the reinforcing fiber fraction, increased moisture retention, and impaired mechanical integrity. Compared with the UF-bonded control, the SCG–ALS composites exhibited lower peak bending strength but significantly higher residual strength after deformation, indicating a more ductile failure mode supported by hydrogen and lignin bonding networks. Thermal analysis confirmed their predominantly organic composition, with lower inorganic residue (2.88–8.46%), delayed combustion, and reduced heat flow relative to UF panels, consistent with the flame-retardant effect of lignosulfonates.

From an application perspective, SCG–ALS biocomposites fulfilled the requirements for particleboard type P2 (EN 312) and, at 50% binder content, also satisfied the MOE criterion for general-purpose MDFs (EN 622-5). However, their internal bond (IB) strength did not meet EN 622-5 requirements, which represents a major limitation for structural or load-bearing applications. This challenge underscores the need for further optimization, such as chemical modification of ALS, incorporation of crosslinkers, or adjustments to processing parameters.

At the same time, the composites may be considered for non-structural, value-added applications where high internal bond strength is not essential. Such potential uses would allow the environmental benefits of SCG valorization to be realized while avoiding direct competition with structural MDFs.

Beyond performance considerations, this study demonstrates the potential of integrating waste-derived resources into engineered wood products. Valorizing SCG as a lignin-rich adhesive component not only diverts agro-industrial waste from disposal but also reduces reliance on fossil-based resins. In combination with lignosulfonates, SCG contributes to a circular, bio-based adhesive system that lowers environmental impact and supports the transition toward sustainable materials in the wood composites industry.

Future research should focus on surface modification of SCGs, chemical functionalization of LS, or the addition of bio-based crosslinkers such as furfuryl alcohol. Such strategies could further enhance mechanical performance and moisture resistance while preserving the formaldehyde-free and sustainable character of the binder system.

## Figures and Tables

**Table 1:** Experimental plan.

Biocomposite Type	SCG Content, %	ALS Content, %	UF Resin Content
A	20	20	0
B	25	25	0
C	30	30	0
D	35	35	0
E	37.5	37.5	0
REF	0	0	10

## Data Availability

The raw data supporting the conclusions of this article will be made available by the authors on request.
